# Identification and validation of a novel immune-related signature associated with macrophages and CD8 T cell infiltration predicting overall survival for hepatocellular carcinoma

**DOI:** 10.1186/s12920-021-01081-z

**Published:** 2021-09-20

**Authors:** Junyu Huo, Liqun Wu, Yunjin Zang

**Affiliations:** Liver Disease Center, The Affiliated Hospital of Qingdao University, No. 59 Haier Road, Qingdao, 266003 China

**Keywords:** Hepatocellular carcinoma, Immune related gene, Macrophages, CD8 T cell, Prognostic signature

## Abstract

**Background:**

Although the effects of macrophages and CD8 T cell infiltration on clinical outcome have been widely reported, the association between immunity-associated gene with them for hepatocellular carcinoma (HCC) remains unclear.

**Materials and methods:**

The ssGSEA served for quantifying the macrophages as well as CD8 T cell infiltration in the HCC samples obtained from TCGA database. Kaplan–Meier (KM) survival assay was used to determine the associations between macrophages and CD8 T cell infiltration with OS. LASSO Cox regressive method assisted in developing an immune gene signature as well as building a risk score. The performance was evaluated by the time-dependent ROC together with the KM survival analysis. The ICGC database were adopted for external verification. CIBERSORT was applied to the correlation analysis on the immune-related signature and the immunocyte infiltration. GSEA were employed exploring the underlying molecular mechanisms.

**Results:**

Increased CD8+ T cell infiltration was associated with longer OS, whereas a greater infiltration of macrophages was related to shorter OS. There were 398 differential expression genes (DEGs) between the high- and low infiltration groups with the “edgeR” package. An prognostic signature consisted of 10 immune genes was built in TCGA and examined in ICGC. The uniform cutoff (0.927) was adopted for separating sufferers into the high-risk (HR) and low-risk (LR) groups. The ROC curves revealed that the AUC data for this signature predicting 1, 2, 3, 4 and 5 year were all above 0.7 in both TCGA and ICGC cohort and patients in the HR group exhibited an evidently weaker prognostic results compared with the LR group. The HR group presented evidently greater Tregs and Macrophage M0 relative to the LR group, whereas the LR group saw the enrichment of CD8 T cells.

**Conclusion:**

The immune signature associated with macrophages as well as CD8 T cell infiltration has reliable prognostic and predictive value for HCC patients.

## Background

Hepatocellular carcinoma (HCC) is a typical primary hepatoma, occupying 85–90% of the total number. It ranks six among all the malignant tumor types worldwide, and the mortality is the fourth highest [1]. Because of there are no obvious symptoms in the early period of the onset of HCC, the majority of sufferers were in the middle and late period during the diagnosis [2], and missed the best opportunity for treatment, so the survival time was short and the prognosis was poor. Therefore, if HCC can be diagnosed and treated in the early stage, and real-time monitoring of the efficacy and prognosis of HCC can greatly improve the therapeutic effect, which is pivotal for extending the survival period and improve the life quality of HCC sufferers. At present, there are still many deficiencies in the traditional clinical indicators for HCC risk stratification and monitoring, which can not effectively guide patients to individualized targeted therapy. Therefore, it is urgent to develop more reliable methods to evaluate the prognosis of HCC in order to guide clinical individualized treatment.

With the deepening research on the mechanism of HCC, it has been found that HCC is often induced by chronic inflammation, accompanied by immune cells (such as T lymphocytes, macrophages, etc.) through the release of various cytokines or direct killing of target cells to play a role in promoting or anti-tumor [3]. So the immunotherapy of HCC [4] has drawn increasing attention. Tumor-associated macrophage (TAMs), one of the important immune cells in tumor microenvironment, is a group of highly plastic macrophages related to specific pathological environment. It has been proved that macrophages can differentiate into type M1 (classical activation pathway) after stimulation with lipopolysaccharide (LPS) and interferon-gamma (IFN- γ), or into type M2 after stimulation with IL-4 (selective activation pathway), thus playing the role of pro-inflammatory, anti-tumor, anti-inflammatory and pro-tumor. At present, it is recognized that TAMs are mainly M2 type, as the key factor of cancer-related inflammation, they promote tumor growth and metastasis by releasing various cytokines (such as IL-10, TGF- β, IL-8, etc.) to inhibit effective anti-cancer immunity, stimulate angiogenesis and epithelial-mesenchymal transformation [5–7]. Similarly, as the main cells of another kind of tumor-associated immunity, T lymphocytes participate in tumor immune monitoring and immune escape by directly recognizing target cells or releasing various cytokines [8]. CD8 T cells, as the primary anti-tumor cells in tumor infiltrating lymphocytes, could realize the releasing of perforin and granule enzyme B via Fas / FasL pathway through cell contact, or destroy targeted cells via the release of IFN—γ and TNF cytokines [3]. Considering that immunotherapy for HCC at this stage only benefits a few people and drug resistance often occurs [9, 10], it is very important to recognize the influence of CD8 T lymphocytes and TAMs on HCC progression. Currently, the function of abnormally expressed immunity-associated genomes during cancer immunity escape is increasingly becoming a novel orientation of cancer investigation [11]. However, whether and how TAMs and CD8T lymphocytes affect the expression of immune genes has not been reported in HCC.

The study holds the purpose of investigating how abnormal immunogenomic expression associated with macrophage and CD8 T lymphocyte infiltration affects the prognosis of HCC and its potential prognostic value and explore its underlying regulatory mechanism in order to provide reference for future precise therapy of HCC.

## Materials and methods

### Data collection

Our team collected the mRNA sequencing data as well as clinical data of 343 HCC samples whose survival time was ≥ 1 month from The Cancer Genome Atlas (TCGA) database. Our team derived 2498 confirmed genes related to immune from the ImmPort database (https://immport.niaid.nih.gov)) [12]. Another independent cohort contained 228 HCC sufferers with complete clinical information and mRNA sequencing results were obtained from the International Cancer Genome Consortium (ICGC) database. The sequencing data of the two databases were all based on lumina platform. What we need to declare is that the acquisition and use of the aforementioned data were completely comply with the rules and regulations of the corresponding database [13]. Because of the data were originated from publicly open database, our research doesn't have to be accepted by the regional ethical board [14].

### Correlation analysis of immune infiltration and prognosis

We used the the single sample Gene Set Enrichment Analysis (ssGSEA) for quantifying the activity or enriching levels pertaining to immunocytes in the HCC samples [15]. The enrichment score represented the abundance exhibited by each immune cell type in the ssGSEA analysis [16]. During the process of immune infiltration estimation, we used R package “GSVA” and “limma” and “GSEABase”. The median value of immune cell infiltration abundance was adopted for separating sufferers into high infiltration and low infiltration groups initially, afterwards, our team used KM survival assay to observe whether it has prognostic significance in HCC. For the variables with *p* < 0.05 tested by log-rank, we further used X-title software to determine the optimal cut-off value [17].

### Identification of immunity-associated DEGs

We used R package"edgeR" to determine the immune genes differentially expressed between low and high abundance (considering the optimal cut-off value under X-title software) of macrophages and CD8 T lymphocytes respectively, TMM (trimmed mean of m-values) approach was employed for normalization, and the function used was “calcNormFactors”, fdr < 0.05 were considered to be of significance.

### Screening of prognostic immune genes

We integrated cox and Kaplan–Meier (patients were sub-classified into group with low expression and group with high expression considering the median gene expression level) survival analysis to identify immune genes related to prognosis. If the p value was less than 0.05 (*p* < 0.05) in both methods, the gene could be considered to affect the prognosis significantly.

### The prognostic model construction in TCGA cohort

The LASSO regression analysis supporting tenfold cross-validation and 1000 bootstrap samples were carried out in this research to remove over-fitting regarding genes related to prognostic firstly using the “glmnet” R package, Subsequently, multivariable Cox regressive method helped to establish the prognostic model [18]. The medium risk score was considered a criterion to classify sufferers into the high-risk (HR) and low-risk (LR) groups [19]. Kaplan–Meier survival curves served for survival analysis and Log Rank test served for statistical analyses. The assessment of the forecast accuracy of the prognosis pattern was conducted via the time-dependent ROC curve produced via R package “survivalROC”. To compare risk score differences among diverse clinical variants, Kruskal–Wallis tests were used. *p* < 0.05 is considered significant on statistics.

### Validation of the general clinical applicability for the prognostic model

For the purpose of the general applicability validation, the 343 sufferers were separated into 26 subgroups according to the different clinical features. The Kaplan‐Meier survival curves served for analyzing the difference in survival between the HR and LR groups in each subgroup.

### Independent verification of the prognosis model

Univariable and multivariable Cox models assisted in examining the independent prognostic value possessed by the risk score and estimating its hazard ratios. *p* < 0.05 is considered significant on statistics.

### Exterior verification of the prognosis model in ICGC cohort

The independent data set (ICGC, n = 228) was adopted for the exterior verification of the prediction effect imposed by the model. KM survival assay together with ROC curve assay served for evaluating the prognostic value.

### Immune infiltration analysis between the high- and low-risk groups

For estimating the relative proportion occupied by 22 infiltrated immune cell types in tumor tissues, we utilized CIBERSORT algorithm to calculate immune cell composition based on normalized expression profiles [20]. For comparing the immune infiltration between the HR and LR groups, Wilcoxon rank-sum test was completed via R function Wilcox test. *p* < 0.05 is considered significant on statistics.

### Gene sets enrichment analysis

Gene Sets Enrichment Analysis (GSEA) assisted in investigating the potential mechanism of risk score in HCC prognosis using GSEA software (v 4.0.1). Our team adopted h.all.v7.1.symbols.gmt as reference gene sets [21].

### Establishment of the survival predictive nomogram

A nomogram was established by the “rms” R package, and ROC curves together with calibration plots assisted in assessing the ability of the nomograph to forecast the OS of HCC.

### Statistical assay

Wilcoxon signed rank test was employed to contrast the successive variates between the 2 groups of independent samples. The continuous variables among multiple groups (> 2) of independent samples contrasted by the Kruskal Wallis test. *p* value < 0.05 was deemed as significant on statistics.

## Results

### Prognostic significance of macrophages and CD8 T lymphocyte infiltration

**Fig. 1:**
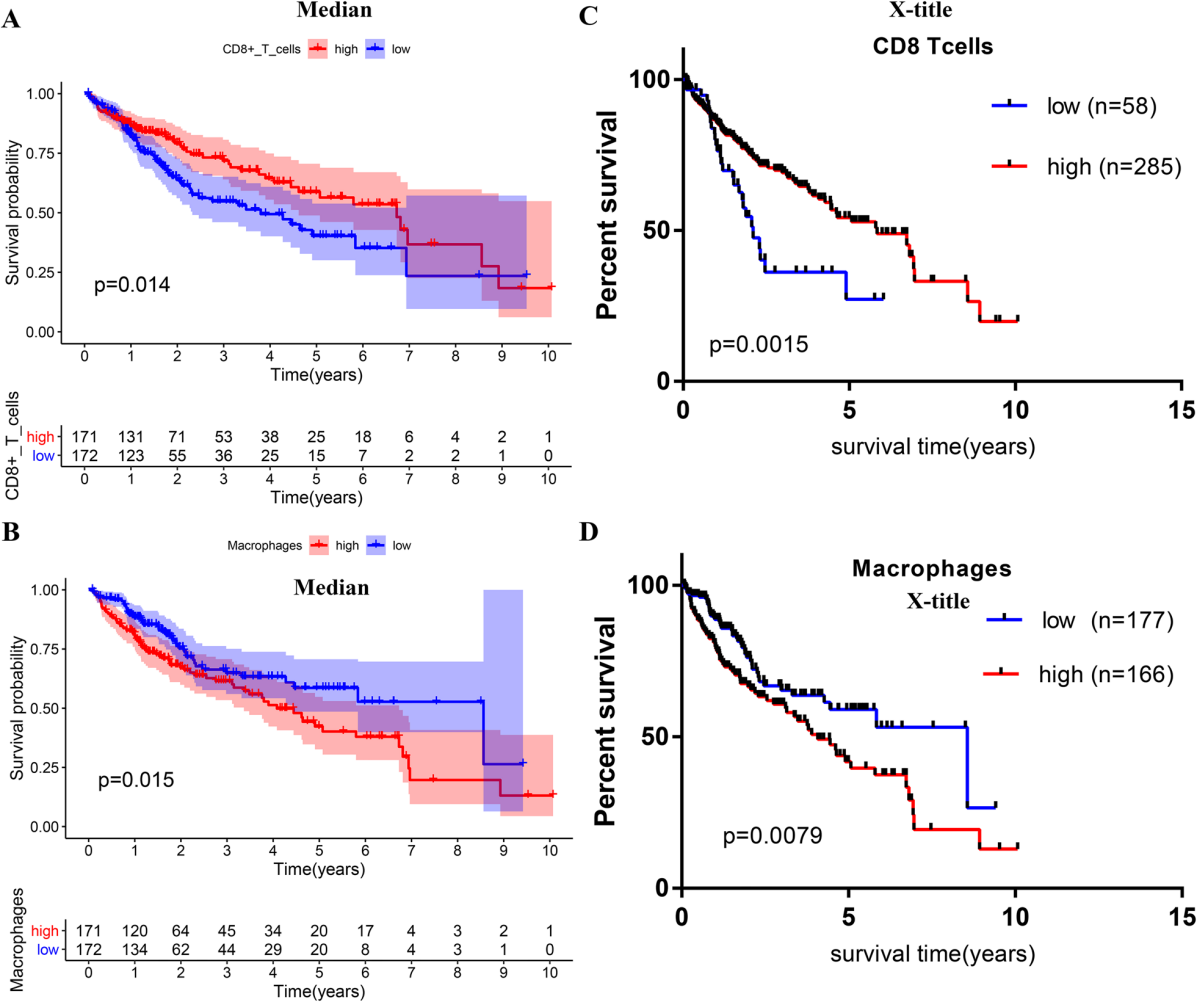
The Kaplan–Meier survival analysis of high CD8 T cell infiltration and high macrophage infiltration for HCC (**a**, **b**). The median value of immune cell infiltration abundance was taken into account for dividing patients into group with high infiltration and group with low infiltration (**c**, **d**). Further used X-title software to determine the optimal cut-off value

The degree regarding macrophages as well as CD8 T lymphocyte infiltration is correlated with the overall survival of HCC. The medium risk scoring determined the cut off values (Fig. 1a, b). Then X‐title software divided patients into the high and low groups, and HCC patients with high infiltration of macrophage presented a weaker prognosis relative to those with low infiltration, while HCC patients with high infiltration of CD8 T lymphocyte exhibited a better prognosis relative to those with low infiltration group (Fig. 1c, d).

### Differential immune related genes (DIRGs) expressed between high and low infiltration groups

**Fig. 2:**
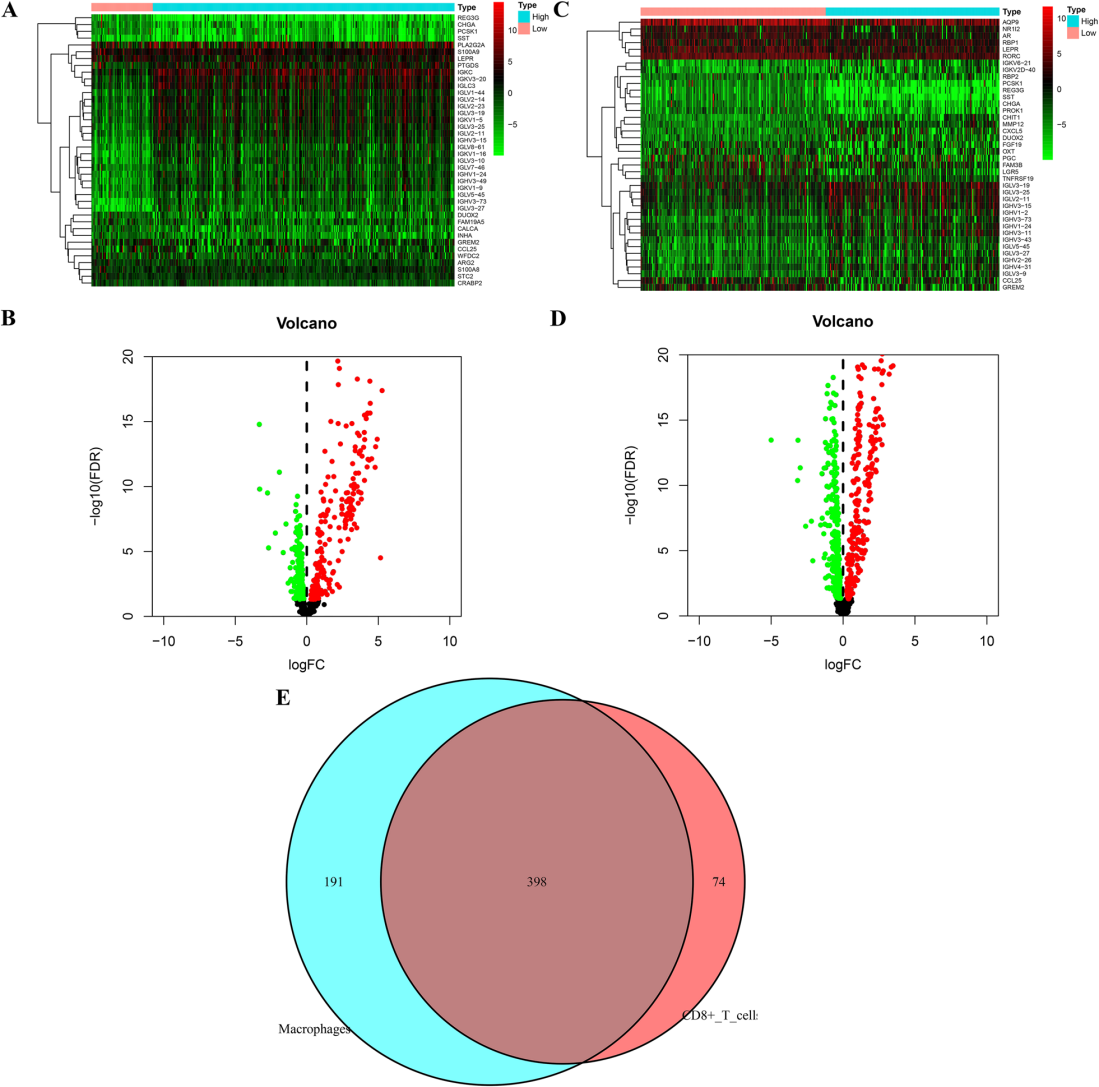
Identification of differential expressed immune-related genes(DEIRGs) between high- and low- immune cell infiltration groups (**a, b)**. The heatmap and volcano map of DEIRGs between high- and low- CD8 T cell infiltration groups (**c**, **d**). The heatmap and volcano map of DEIRGs between high- and low- macrophages infiltration groups (**e**). The Venn plot of intersection DEIRGs

As identified herein, 208 genes showed significant upregulation and 264 genes showed significant downregulation in the CD8 T lymphocyte high-infiltration tumor tissues with *p* value < 0.05 after FDR adjustment (Fig. 2a, b). There were 261 genes that exhibited significant upregulation and 328 genes that exhibited significant downregulation in the macrophages high-infiltration tumor tissues with *p* value < 0.05 after FDR adjustment (Fig. 2c, d). Altogether, 398 genes were obtained in the intersection of the aforementioned two groups of differential -expressed genes. (Fig. 2e).

### Screening of immune genes related to prognostic results

**Fig. 3:**
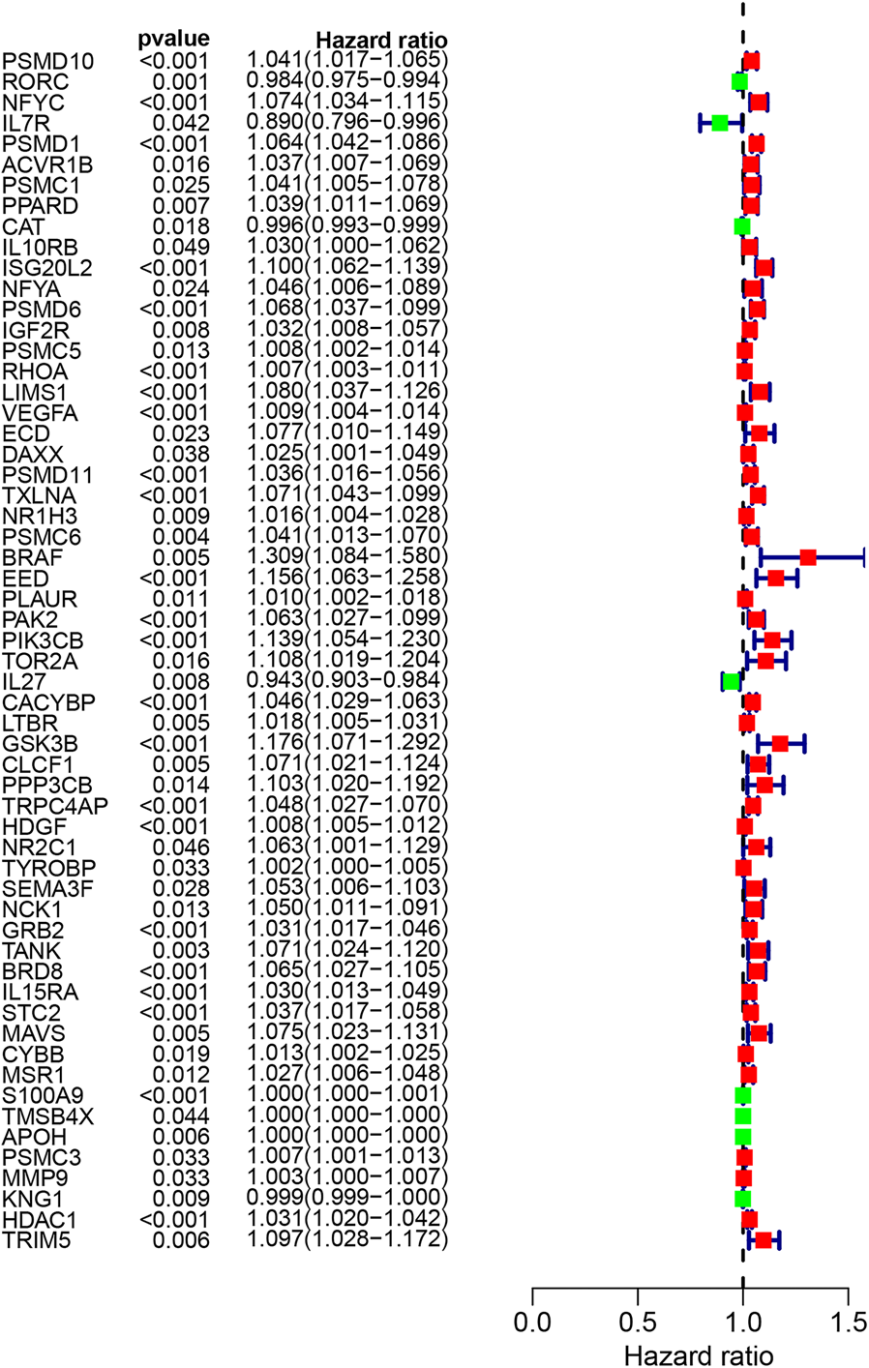
The forrest plot of prognostic DEIRGs identified by univariate Cox and Kaplan–Meier survival analysis

To determine the DIRGs with prognosis significance, the above 398 genes were evaluated by univariable Cox and and KM survival assay. After screening, we selected 58 genes of which 52 were risk factor and 6 were protective factors (Fig. 3).

### Establishment of the 10-immune gene signature

**Fig. 4:**
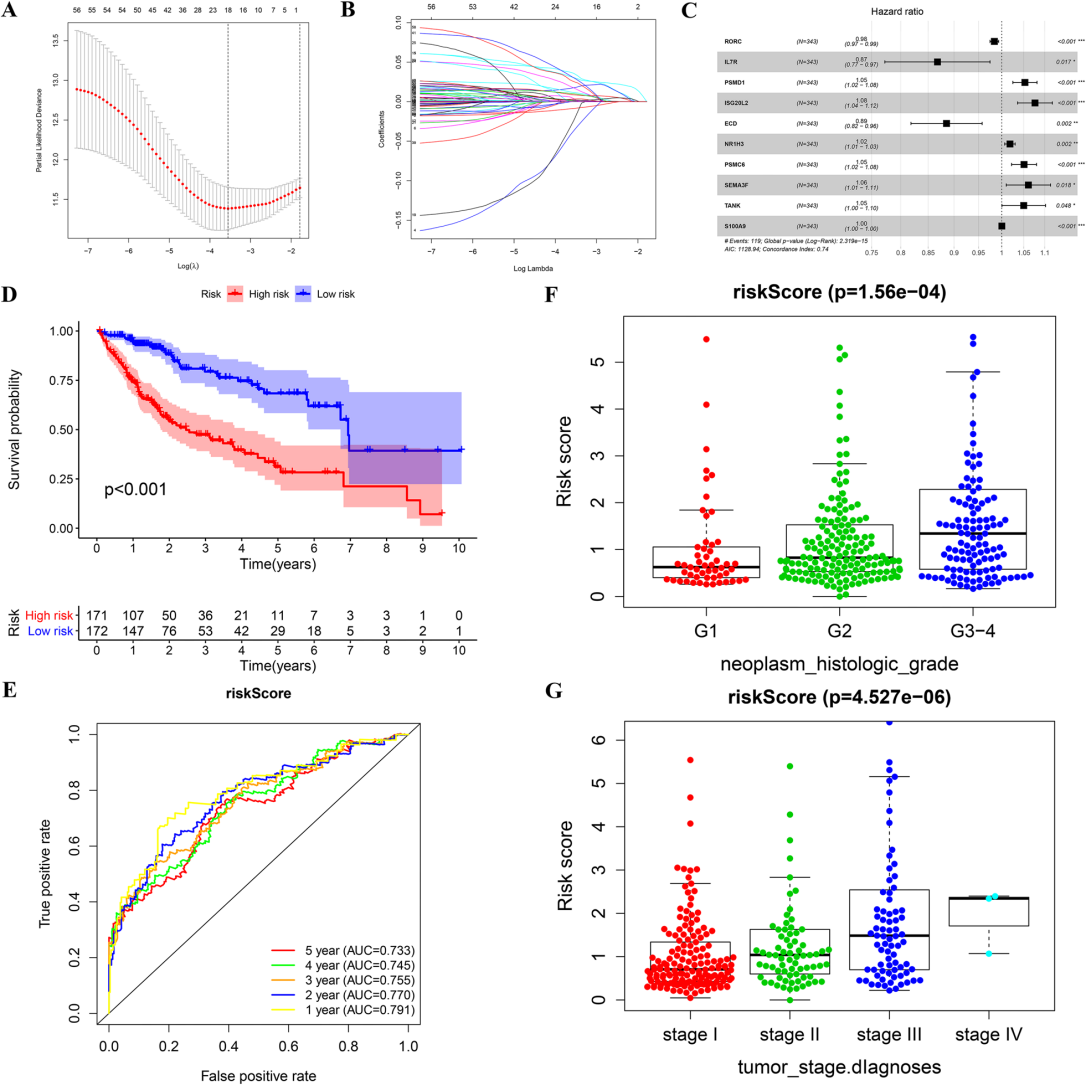
The Establishment of the 10-immune gene signature (**a**–**c**). The establishment of the prognostic model based on Lasso penalized Cox analysis (**d**–**e**). Kaplan–Meier survival analysis and time-dependent ROC analysis of predicting overall survival for patients in TCGA cohort used by risk score (**f**). The relationship between risk score and histologic grade(Kruskal–Wallis test) (**g**). The relationship between riskscore and TNM grade(Kruskal–Wallis test)

To identify the core genes for predicting prognosis, Lasso and multivariable Cox regressive assay helped to detect which genes were independence prognosis factors for the OS in the TCGA cohort (Fig. 4a–c). 58 genes related to prognostic immune received Lasso Cox analysis, with 10 genes being filtered out (Fig. 4c). The formula specific to the risk score was presented in Table1. The median of risk score (0.927) was considered as a standard to divide patients into the HR and LR groups. The KM survival assay revealed that the HR group exhibited an evidently worse OS compared with the LR group (*p* < 0.001) (Fig. 4d). To evaluate the prognostic model, the 1–5 year ROC curves were plotted and the C-index was computed. As shown in Fig. 4c, The C‐index was 0.74 for the prognostic model. The AUC registered 0.791, 0.770, 0.755, 0.745, and 0.733 at 1, 2, 3, 4, and 5 year respectively, indicated good accuracy of this model (Fig. 4e). We also evaluated the association between risk score and tumor stage, as well as with tumor histological grade. As shown in figure F–G, risk score showed an obvious relation to higher histologic grade (*p* < 0.001) and late period (*p* < 0.001).

**Table 1:** The list and coef of the 10-gene signature

Gene name	coef	HR	HR.95L	HR.95H	pvalue
RORC	−0.0162	0.983933	0.974606	0.99335	0.00086
IL7R	−0.14209	0.867547	0.772266	0.974584	0.016681
PSMD1	0.05136	1.052701	1.024906	1.08125	0.000169
ISG20L2	0.073621	1.076399	1.035809	1.11858	0.000174
ECD	−0.122	0.885147	0.818185	0.957589	0.002368
NR1H3	0.018322	1.018491	1.006811	1.030307	0.00185
PSMC6	0.049424	1.050666	1.022064	1.080068	0.000448
SEMA3F	0.059062	1.060841	1.01014	1.114086	0.018091
TANK	0.04874	1.049947	1.000512	1.101825	0.047616
S100A9	0.000414	1.000414	1.000184	1.000644	0.000419

### General applicability verification of the prognosis signature

**Fig. 5:**
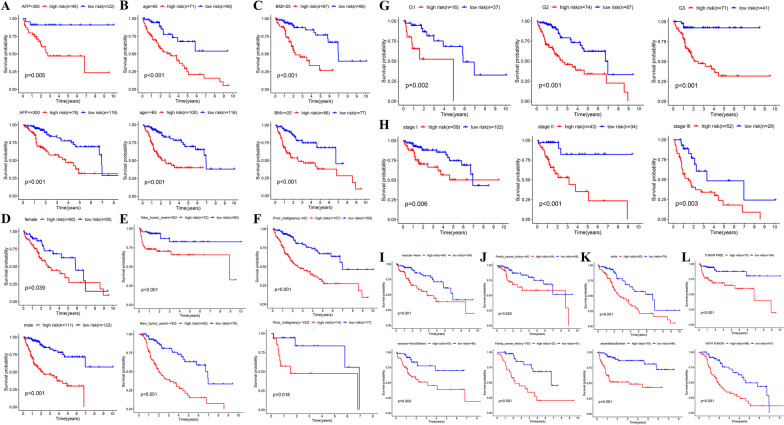
Internal validation of the prognostic model based on different clinical features of HCC patients in TCGA cohort (Kaplan–Meier survival analysis). **a** AFP. **b** Age. **c** BMI. **d** Sex. **e** New tumor event after initial treatment. **f** Prior malignancy. **g** Histology grade. **h** Stage TNM. **i** Vascular tumor cell type. **j** Family cancer history. **k** Race. **l** Personal cancer status

For determining if the prognostic model applied to HCC patients with different clinical features, we performed further analyses in 26 subgroups in order to ascertain the robustness of our findings. As revealed by the survival analysis, HCC patients in the HR group exhibited a poor prognostic result compared with the LR group, and this outcome was observed in each subgroup (Fig. 5a–l).

### Validation of the independent prognostic value possessed by the prognostic signature

**Fig. 6:**
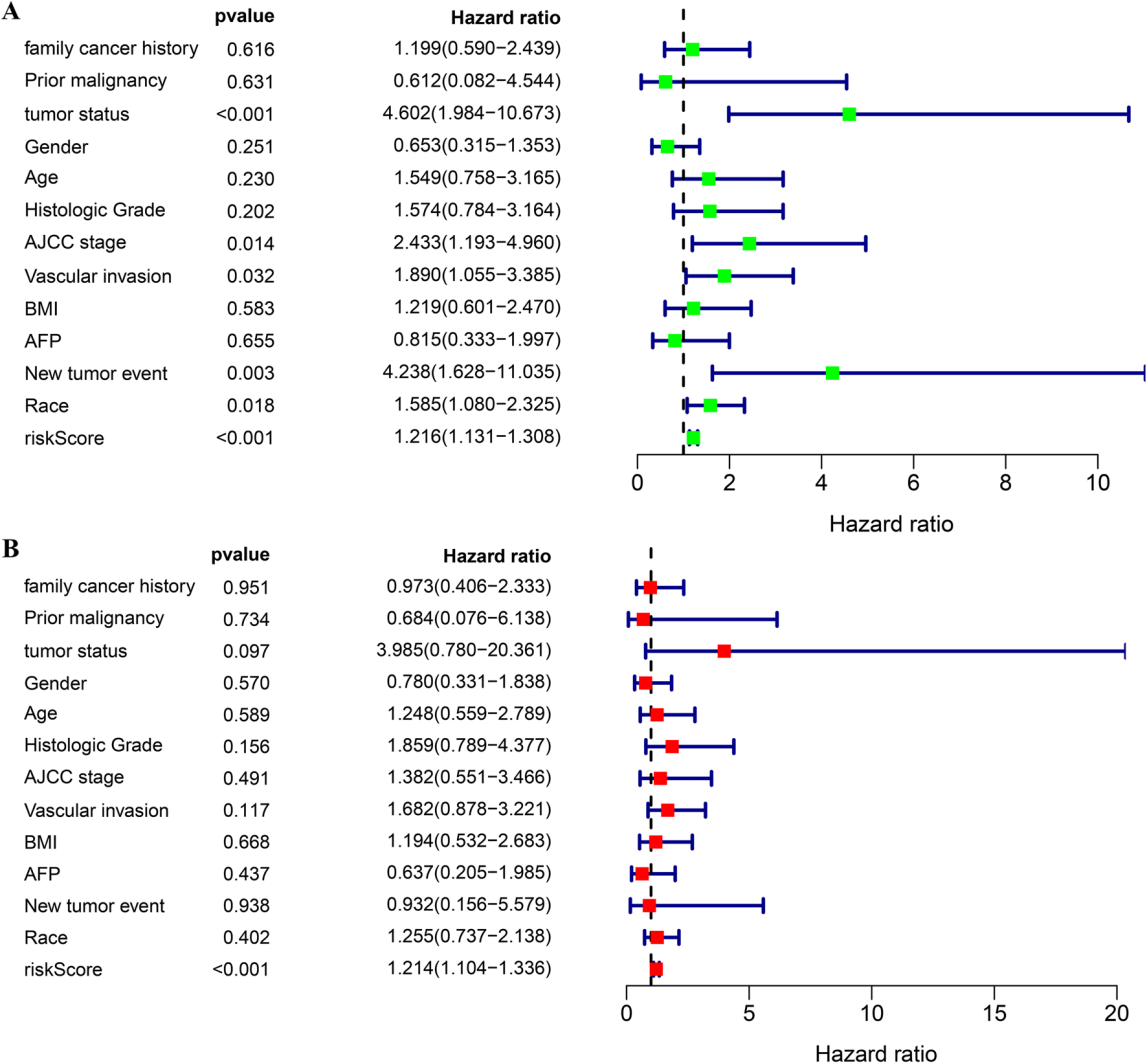
Independence validation of the risk score for predicting overall survival of HCC in the TCGA cohort. **a** Univariate Cox analysis. **b** Multivariate Cox analysis

Univariable and multivariable Cox regressive assay of the signature and other -clinicopathological variables showed that the risk scoring could serve for the independence prediction of the HCC prognostic results (Fig. 6a, b).

### Exterior verification of the prognosis signature in ICGC cohort

**Fig. 7:**
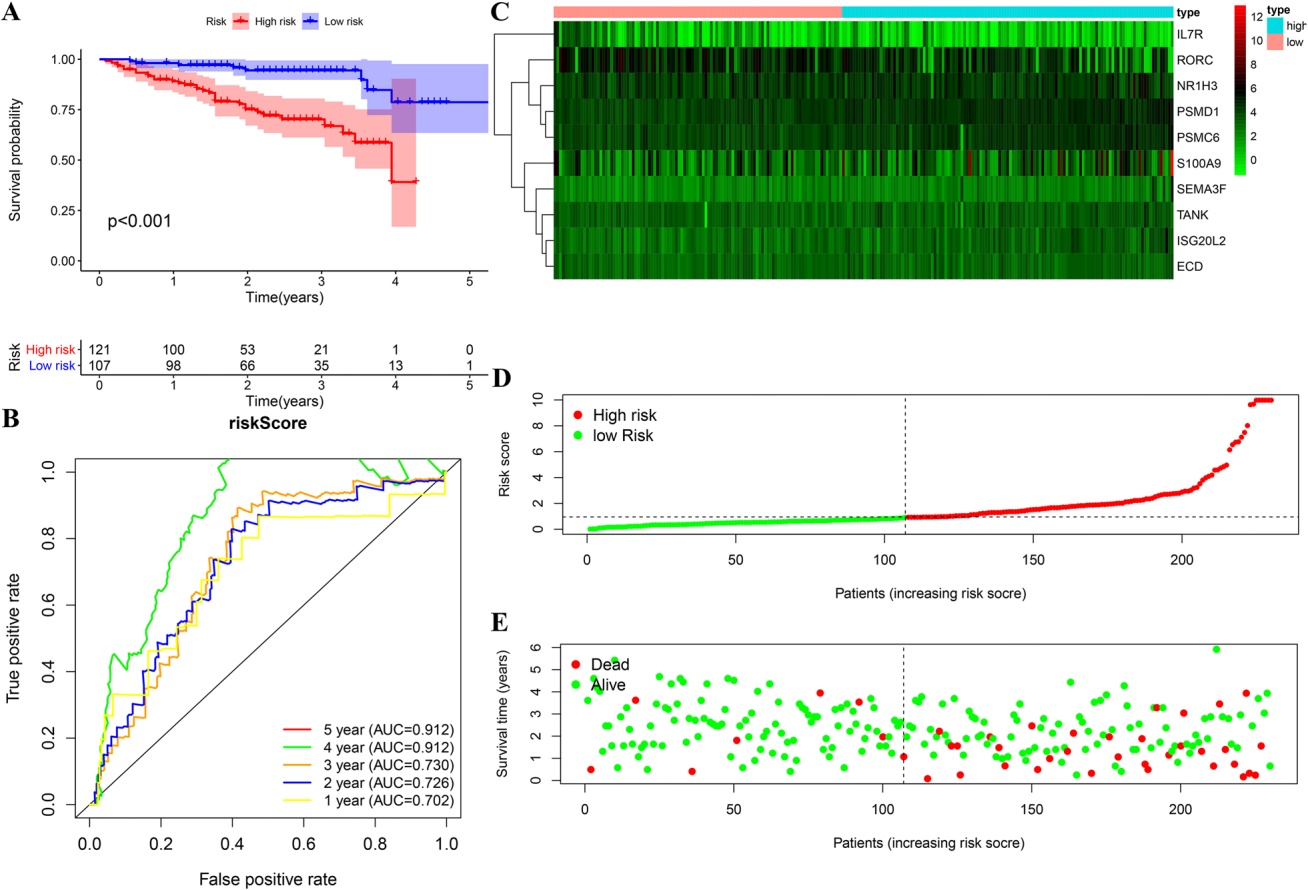
External validation of the prognostic model in ICGC cohort (**a**, **b**) Kaplan–Meier survival analysis and time-dependent ROC analysis of predicting overall survival for patients in ICGC cohort used by risk score (**c**–**e**). The heatmap of the 10-gene signature and the distribution of risk score and the survival status of patients

Our team computed the risk scoring of all patients based on the 10-immune gene signature and stratified sufferers into the HR and LR groups considering their risk score using the identical cutoff as for the TCGA cohort. As seen from the Kaplan–Meier survival curve, the OS difference regarding the two groups exhibited a statistical significance (*p* < 0.001) (Fig. 7a). Consistent with the TCGA results, patients in the HR group had a shorter OS compared with those in the LR group. ROC analysis illustrated an AUC ROC curve specific to 1-, 2-, 3-, 4-, and 5-year survival prediction by the 10-gene signature were 0.702, 0.726, 0.730, 0.912, and 0.912 respectively (Fig. 7b). Low-risk patients were found to have lower death rates and longer survival times than those in the high-risk patients (Fig. 7c–e).

### Analyses of the immune cell infiltration and immune function between different risk groups

**Fig. 8:**
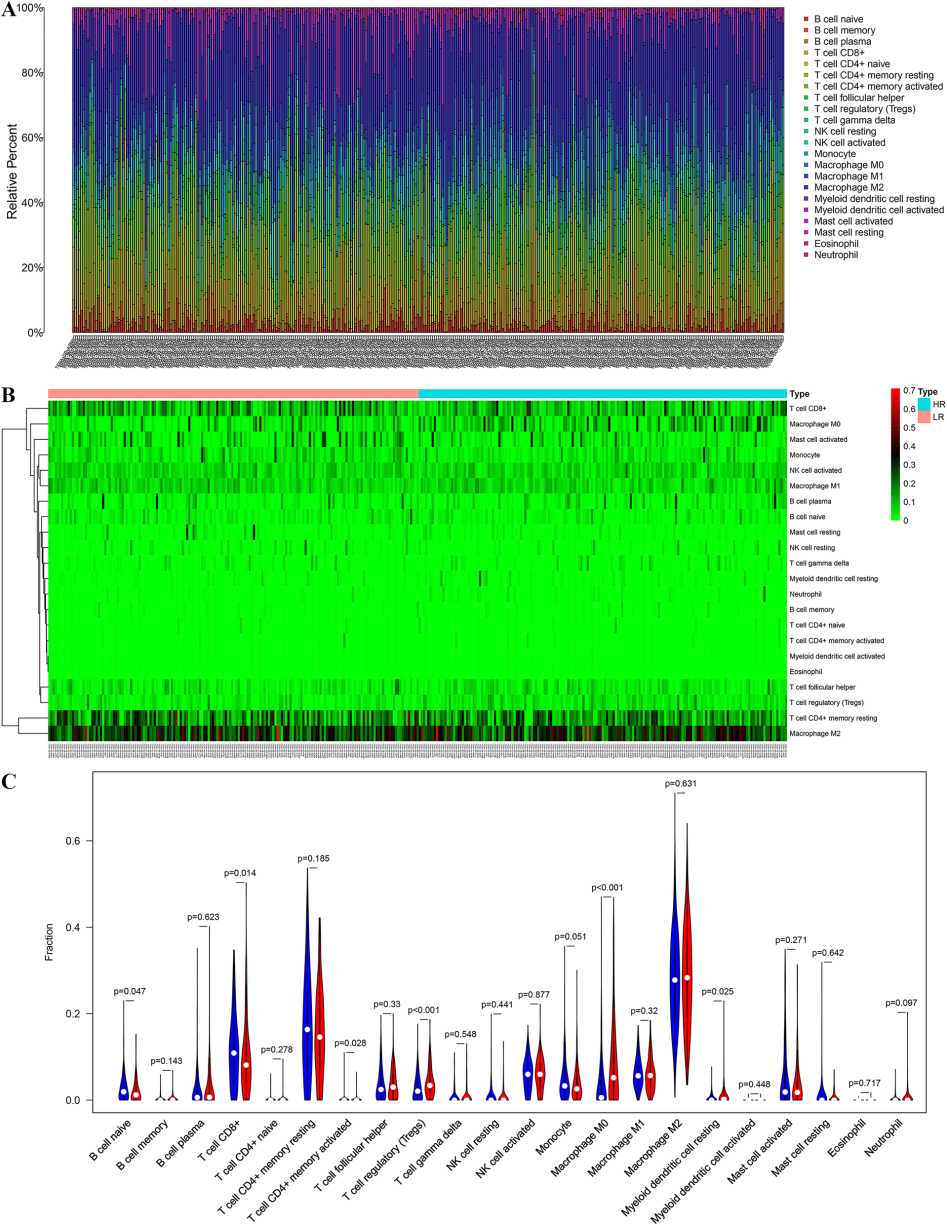
The immune infiltration landscape in HCC patients with high- and low-risk utilized CIBERSORT algorithm. **a** The barplot of the proportion of immune cell infiltration. **b** The heatmap of the proportion of immune cell infiltration. **c** The violin plot of 22 types of immune cell infiltration abundances in groups with high and low risks (Wilcoxon signed-rank test, red and blue denote high and low risk)

**Fig. 9:**
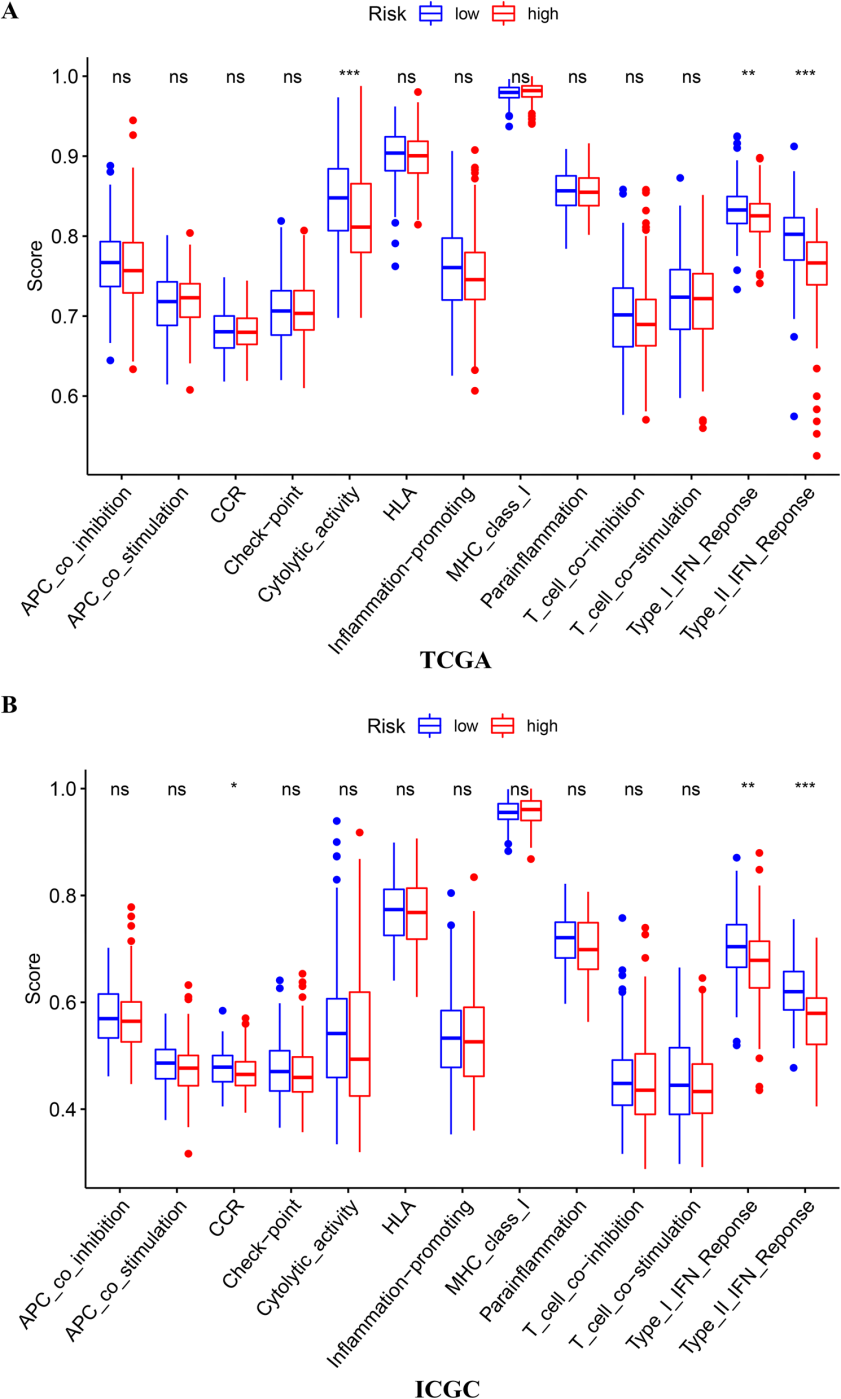
The immune function differences between high- and low-risk HCC patients estimated by ssGSEA algorithm(Wilcoxon signed-rank test). **a** The boxplot of 13 types of immune-function score in groups with high and low risks of TCGA cohort. **b** The boxplot of 13 types of immune-function score in groups with high and low risks of ICGC cohort

B cell naive and CD8 T cells infiltration was in an obviously higher level in LR tumors relative to HR tumors (*p* < 0.05), while infiltration of macrophage M0 and Tregs in HR tumors was in an obviously higher level compared to LR tumors (Fig. 8a–c). In terms of immune function, Type I and Type II IFN reaction in the HR group were all more remarkable versus the LR group in both TCGA and ICGC cohort (Fig. 9a–b).

### GSEA of the prognostic signature

**Fig. 10:**
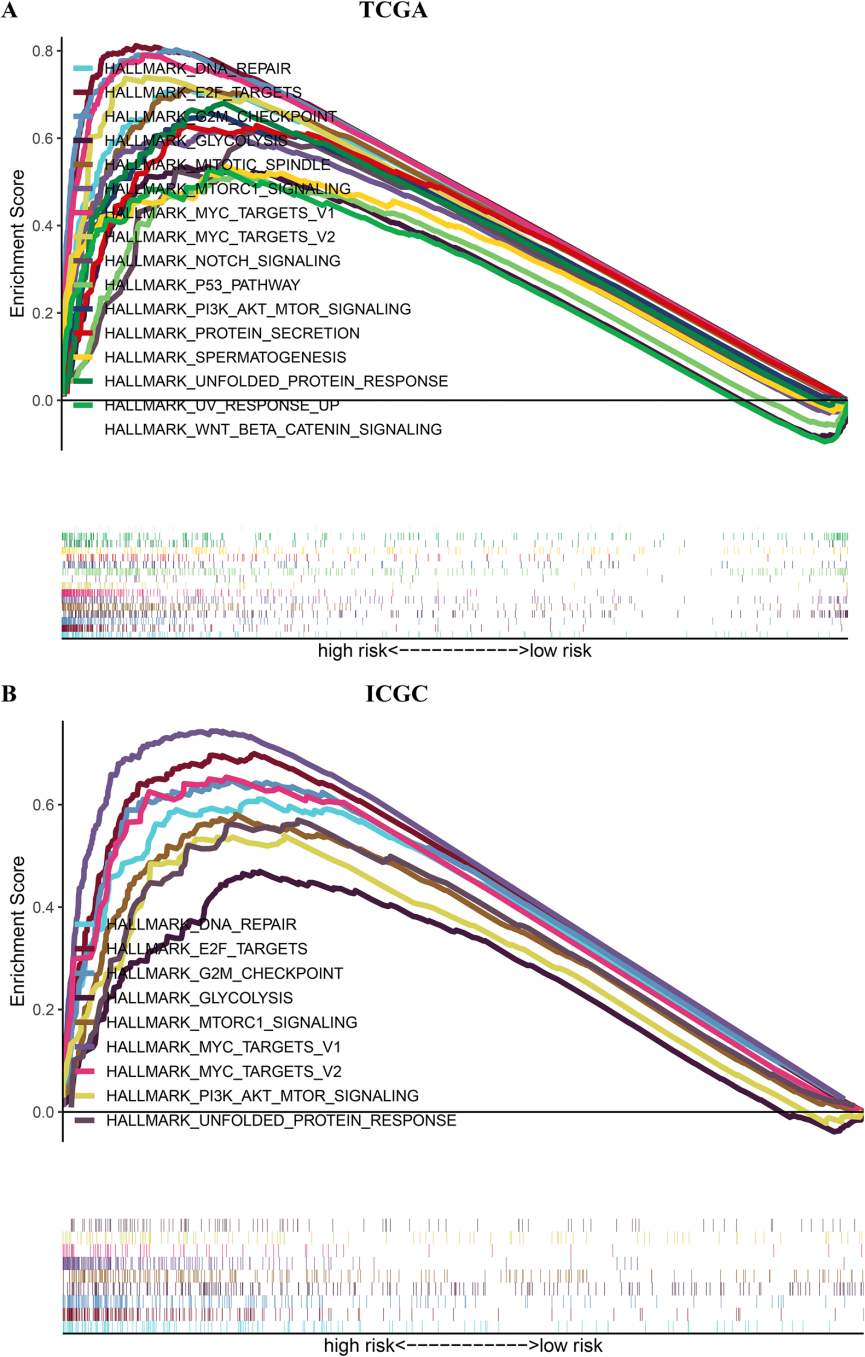
Gene Set Enrichment Analysis between different risk groups. **a** TCGA cohort. **b** ICGC cohort

GSEA was conducted to compare samples in different risk groups, aiming at investigating the underlying mechanisms that caused different clinical outcomes. As found, the gene set enriched in group with a high risk involved in many aspects of the occurrence and development of oncology, such as DNA restoration, glucolysis, MYC targets, PI3K-AKT-MTOR signal path, etc. (Fig. 10a, b).

### Building a nomogram for overall survival prediction

**Fig. 11:**
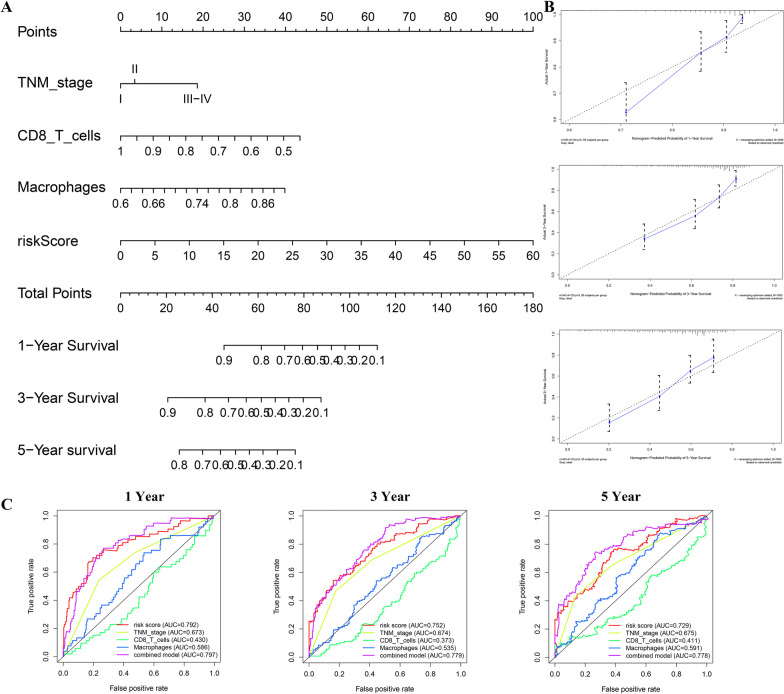
Development and validation of a nomogram predicting OS for HCC. **a** Nomogram for predicting the probability of 1-, 3-, and 5-year OS for HCC patients. **b** Calibration plot of the nomogram for predicting the probability of OS at 1, 3, and 5 years. **c** Time-dependent ROC curve analyses of the combined prognostic model and other prognostic factors

The nomogram of a combined model was built, which contained TNM stage, CD8 T cells, macrophages and risk score (Fig. 11a). According to the calibration curve, the 1-, 3-, and 5-year OS prediction under nomogram and the actual probabilities matched reasonably well (Fig. 11b). ROC curve showed that the nomogram could effectively make up for the limitation of single prediction factor (Fig. 11c).

## Discussion

Immune escape is known as one of the hallmarks of cancer [22, 23], therefore, anti-tumor immune activation has become one of the hot spots in oncology studies recently. CD8 T cells and macrophages are two major immune cells infiltrating the tumor microenvironment, which have been reported to be related to the efficacy of tumor immunotherapy [24]. However, we have not yet found a definite answer to whether CD8 T cells and macrophages will affect the prognosis of HCC, and what mechanisms are involved in it. Herein, our team linked the level of immunocyte infiltrative activity with the immunity gene expression, trying to explore the prognostic mechanism of HCC from a novel standpoint.

In this study, we conducted a comprehensive and detailed assessment of immune infiltration in HCC using the operation of ssGSEA algorithm, our team discovered that the elevated CD8 T cells infiltration were correlated with favorable prognosis, while a high degree of macrophages infiltration implied poorly prognosis, no matter which method (the median value or the best cutoff from x-title) was used for dividing patients into different groups. Considering the mechanisms behind this phenomenon were still unknown, we extracted immune-related genes from ImmPort, trying investigated it from immune gene perspective further. We found that when the optimal cutoff from x-title was selected to divide the high—and the low infiltration group, the diversity in prognostic results between the 2 groups were most striking. In subsequent research, we used the optimal cutoff from x-title for dividing groups with high—and the low infiltration and detected the immunity-associated DEGs between the 2 groups. Following univariable Cox and and KM survival analysis, 58 genes obviously associated with prognosis were screened from 398 intersection differentially expressed immune-related genes. Furthermore, LASSO regressive analyses and multivariable Cox regressive analysis identified 10 key mRNAs, which were used to establish the prognostic model. We acquired the individualized risk score of patients based on our scoring system. The medium risk scoring of TCGA cohort was taken as the uniform cut-off for classifying HCC sufferers into the HR and LR groups. The survival curves indicated that patients in the HR group showed an evidently shorter OS compared with the LR group. The performance of the risk scoring was assessed by the ROC curves, showing that this predictive model effectively predicted HCC patients’ OS, with all AUC values greater than 0.7. The risk score also showed a positive relation to the tumor stage and grade in HCC, a high risk score is may reflect a higher degree of malignancy. Further analysis was conducted on patients with different clinical features. As indicated by the survival analysis, the HR group exhibited a weaker prognostic result relative to the LR group in all 26 subgroup. As per the univariable and multivariable Cox regressive assay, the risk score model could serve for the independent prediction of the prognostic results.

Next, we validated our model externally in ICGC cohort and demonstrated good performance. As revealed by the survival curves, the HR and LR groups divided based on the signature exhibited different prognoses. As per the ROC curves, the AUC data for our signature forecasting OS at 1, 2, 3, 4, and 5 year were all greater than 0.7.

To explore the mechanism of the predictive signature, we performed both GSEA and CIBERSORT immune infiltration estimation. GSEA demonstrated that gene sets related to PI3K-AKT-mTOR pathway, glycolysis and DNA repair, etc. presented a positive enrichment in the HR group of both TCGA and ICGC cohort. In terms of immune infiltration, the HR group saw an evidently larger proportion of macrophagus M0 and Tregs and in the LR group, B cells and CD8 T cells were significantly and highly enriched. All these findings helped us to better understand the mechanism of HCC. Finally, in order to improve the model prediction accuracy, we incorporated risk score, CD8 T cells, macrophages and AJCC-TNM stage into the predictive nomogram, which made our research more quantitative and intuitive.

The molecular mechanism of several genes in our signature has been reported previously in HCC. For example, Kong demonstrated the upregulation of Interleukin-7 receptor (IL-7R) could facilitate the proliferative and migratory activities of liver cancer cells through NF-κB and Notch1 pathways [25]. Tan found that PSMD1 regulate the cellular lipid metabolism via p38-JNK and AKT signaling, which regulate HepG2 cells proliferation [26]. Duan found that blood serum and tissue samples from HCC patients who were HBV-positive possessed a higher S100A9 expression relative to those who were HBV-negative, and the silencing S100A9 expression to some extents blocked the HepG2 cell growth and metastasis induced by HBx in vitro and in vivo [27]. The exact role of other genes in HCC is still elusive.

Herein, we linked the expressing of immunity genes with the infiltration of immune cells and analyzed their prognostic significance for HCC. Secondly, we developed one model using various statistical methods and performed internal validation. Moreover, the robustness of our final model was demonstrated by exterior verification, which is a vital for the clinic application of a model. We finally established an nomogram consisted of TNM stage, CD8 T cells, macrophages as well as risk score, which is convenient for clinicians to use. As a retrospective study, main limitations of the present study derive from its retrospective nature, hence, it's imperative to conduct a forward looking research with multiple centers in the future. In addition, further experiments shall be conducted for elucidating the mechanisms related to the signature.

## Conclusion

The study provided a new tool to evaluate the prognosis of HCC, which from the perspective of macrophage and CD8 T cell infiltration.

## Data Availability

The datasets analysed in this research were downloaded from The Cancer Genome Atlas (TCGA, https://portal.gdc.cancer.gov/repository) and International Cancer Genome Consortium (ICGC, https://dcc.icgc.org/releases/current/Projects/LIRI-JP). The original data for our study were available on https://datadryad.org/stash/share/uH9cXPBu-VmH52RIZGUCgPjXsYob6gh8HTuBooWQntg.

## Abbreviations

HCC: Hepatocellular carcinoma
TCGA: The Cancer Genome Atlas
ICGC: International Cancer Genome Consortium
DEGs: Differential expressed genes
DIRGs: Differential immune-related genes
GSEA: Gene Set Enrichment Analysis
ssGSEA: Single sample Gene Set Enrichment Analysis
GSEA: Gene Set Enrichment Analysis
KM: Kaplan–Meier
TMM: Trimmed mean of m-values
ROC: Receiver operating characteristic
AUC: Area under curve
OS: Overall survival
LASSO: Least absolute shrinkage and selection operator
FDR: False discovery rate
HR: High-risk
LR: Low-risk
